# Quantifying the effects of hemoglobin saturation on plasma strong ion difference during blood oxygenation and decarboxylation

**DOI:** 10.14814/phy2.71118

**Published:** 2026-09-29

**Authors:** Lorenzo Giosa, Martin Krbec, Jakub Halamík, Adrian Bolnberger, Mattia Busana, Serena Brusatori, Tue Diemer, Søren R. Kristensen, Micah L. A. Heldeweg, Francesco Zadek, Stephen E. Rees, František Duška

**Affiliations:** ^1^ Department of Anaesthesiology and Intensive Care, 3rd Faculty of Medicine Charles University and Kralovske Vinohrady University Hospital Prague Czech Republic; ^2^ Department of Anaesthesia, Intensive Care and Pain Medicine, Medical Clinical Division of General Anaesthesia and Intensive Care Medicine Medical University of Vienna Vienna Austria; ^3^ Department of Anesthesiology University Medical Center of Göttingen Göttingen Germany; ^4^ Fondazione IRCCS Ca’ Granda Ospedale Maggiore Policlinico Milan Italy; ^5^ Department of Clinical Genetics Aalborg University Hospital Aalborg Denmark; ^6^ Department of Clinical Medicine Aalborg University Aalborg Denmark; ^7^ Department of Clinical Biochemistry Aalborg University Hospital Aalborg Denmark; ^8^ Department of Anesthesiology Amsterdam University Medical Centers Amsterdam The Netherlands; ^9^ Department of Medicine and Surgery University of Milan‐Bicocca Monza Italy; ^10^ Institute for Health Science and Technology Aalborg University Aalborg Denmark

**Keywords:** albumin, hemoglobin, non‐carbonic whole‐blood buffer power, oxygen saturation, plasma strong ion difference

## Abstract

The strong ion difference (SID) is assessed to interpret acid–base disorders, yet measured plasma values are influenced by electrolyte redistribution across compartments. Current models focus on pH‐dependent plasma‐erythrocyte shifts. Here, we aim to quantify the effects of hemoglobin oxygen saturation (sO_2_). We induced oxygenation and decarboxylation of human venous blood (*n* = 20) via room‐air equilibration. We modeled the contribution of ∆sO_2_ to plasma‐erythrocyte shifts through the Haldane effect and hypothesized that combining sO_2_‐ with pH‐dependent mechanisms would allow accurate prediction of redistribution related ∆SID. After room‐air equilibration, sO_2_ fraction increased by 0.46 [0.39–0.54], CO_2_ tension decreased by 29 [25–32] mmHg, and pH increased by 0.25 [0.19–0.32]. SID decreased by 5.3 [4.2 to 5.6] mEq/L, and its changes were independently associated with ∆sO_2_ (∆SID/∆sO_2_ = −3.0 [−5.4 to −0.67] mEq/L, *p* < 0.01). Accordingly, when only pH‐dependent redistribution was considered, ∆SID prediction yielded a ∆sO_2_‐dependent (*p* < 0.01) underestimation of measured ∆SID (mean bias [limits of agreement]: −1.6 [−3.7 to 0.5] mEq/L). Including sO_2_‐dependent effects improved the bias (−0.4 [−2.0 to 1.3] mEq/L) and removed its ∆sO_2_‐dependence (*p* = 0.63), and this result was maintained using a simplified model (∆SID = 1.5·[Hemoglobin+Albumin]_g/dL_·∆pH+[Hemoglobin/4]_g/dL_·∆sO_2_). We conclude that ∆sO_2_ independently affects ∆SID during blood oxygenation and decarboxylation. Incorporating sO_2_‐ alongside pH‐dependent electrolyte shifts enables accurate prediction of redistribution‐related ∆SID.

## 1 INTRODUCTION

Blood gases and electrolytes are assessed multiple times a day to guide clinical management in critically ill patients (Pilon et al., 1997). The strong ion difference (SID), defined as the difference between fully dissociated cations and anions, is a key determinant of acid–base status: reductions in SID are associated with metabolic acidosis, increases in SID with alkalosis (Stewart, 1981). In clinical practice, SID is calculated from plasma electrolytes. However, whole blood is a bi‐compartment system, with red cells accounting for a substantial fraction of blood volume and containing a large pool of charges (Wooten, 2003). Importantly, the erythrocyte membrane is permeable to water and selected strong ions, primarily chloride and lactate. Consequently, redistribution of electrolytes between plasma and red blood cells can alter plasma SID without changing the total pool of whole‐blood charges. Such SID variations represent physicochemical redistribution rather than a primary acid–base disturbance, and failure to recognize this mechanism leads to misinterpretation of acid–base status (Giosa, Zadek, et al., 2024).

A quantification of *pH‐induced* electrolyte shifts (Funder & Wieth, 1966; Dalmark, 1975) was previously put forward by our group, leading to a simplified equation that predicts plasma SID variations from albumin and hemoglobin charges, or the whole‐blood buffer power (*β*) (Giosa, Zadek, et al., 2024). By enabling quick and accurate estimations of pH‐induced SID changes, this approach has shown to improve the interpretation of base excess at the bedside (Krbec et al., 2026). However, the current model assumes fully oxygenated blood; therefore, its validity is limited in conditions characterized by changes in hemoglobin oxygen saturation (sO_2_), such as venous blood sampling, extracorporeal gas exchange, or acute alterations in pulmonary gas transfer. Indeed, changes in sO_2_ constitute an additional determinant of plasma‐erythrocyte electrolyte redistribution (Hastings et al., 1924). This phenomenon, known as the Haldane effect (Christiansen et al., 1914), comprises two distinct mechanisms. *First*, oxygen binding to hemoglobin reduces its buffering capacity by lowering the dissociation constant (pK) of some weak amino acid residues; *second*, oxygenated hemoglobin has a reduced affinity for chloride. These combined effects result in the outward movement of chloride and water (Prange et al., 2001; Westen & Prange, 2003; Wolf, 2022). Despite being well recognized qualitatively, the quantitative impact of these sO_2_‐related processes on plasma SID has not been fully characterized.

The aim of the present study is to quantify *sO*_*2*_ *‐dependent* changes in plasma SID and integrate them into our previously proposed predictive model (Giosa, Zadek, et al., 2024). We re‐analyzed data from an in‐vitro human study in which venous blood was equilibrated with room air, therefore undergoing simultaneous oxygenation and decarboxylation (Rees et al., 2012). We hypothesize that SID variations are independently associated with changes in sO_2_, and failure to consider saturation‐dependent effects leads to underestimation of SID changes in proportion to changes in sO_2_. We also propose a simplified predictive model of redistribution‐related plasma SID changes requiring only clinically available variables to facilitate practical application.

## 2 MATERIALS AND METHODS

### 2.1 Study population and procedures

Data were obtained from a previously published study from our group, where full methodological details are provided (Rees et al., 2012). Briefly, in 20 healthy volunteers (9 male and 11 female participants, age range 32–65 years old), venous blood (9 mL) was collected into a heparinized syringe (Monovette, Sarstedt, Germany) and divided into three 3 mL samples. One sample remained undiluted, while the other two were mixed with a diluting buffer solution containing Na^+^ 145 mmol/L, Cl^−^ 120 mmol/L and HCO_3_^−^ 25 mmol/L, with PCO_2_ similar to that of blood achieved by equilibration with 11% CO_2_. Blood‐to‐buffer ratios in diluted samples were 2:1 and 1:1 in 12 subjects and 4:1 and 1:2 in 8 subjects, generating a broad range of hemoglobin and albumin concentrations. After mixing and removal of air, all three samples were analyzed for acid–base variables (ABL800). Subsequently, the remaining blood in each syringe was equilibrated with room air: each syringe was filled with air to a total volume of 10 mL and continuously rotated for 5 min to prevent sedimentation and promote blood‐air gas exchange. The air was then expelled and replaced with fresh room air three times, with continued rotation between exchanges to facilitate equilibration. The whole procedure lasted around 20 min, during which samples were kept at room temperature. Following the final equilibration, all three blood samples were reanalyzed for acid–base variables (ABL800). Finally, the remaining blood was centrifuged and the plasma analyzed for albumin concentration (Immage 800 analyzer). The original study was conducted in accordance with the Declaration of Helsinki, it was approved by the ethical committee of the North Jutland Region of Denmark (reference number N‐20100093), and informed written and oral consent was obtained from all subjects.

### 2.2 Calculations

In all equations, the subscripts “pre” and “post” refer to blood taken before and after air aspiration. The symbol ∆ refers to the difference between post and pre values.

SID was calculated from measured plasma electrolytes in mEq/L:

(1) $$\mathrm{SID}=\left[{\mathrm{Na}}^{+}\right]+\left[K^{+}\right]+\left[{\mathrm{Ca}}^{2+}\right]-\left[{\mathrm{Cl}}^{-}\right]-\left[{\mathrm{Lac}}^{-}\right]$$

where Ca^2+^ refers to ionized calcium.

The pH‐dependent charge on albumin and hemoglobin (*Z*_pH_) was computed as the sum of albumin + hemoglobin titrable charges, both calculated as (Giosa, Zadek, et al., 2024):

(2) $$Z_{\mathrm{pH}}=c\cdot \frac{n\cdot 10^{-\mathrm{pH}}}{10^{-6.75}+10^{-\mathrm{pH}}}$$

where *c* is the protein concentration (mMol/L), *n* is the number of its titrable groups, and 6.75 is their average dissociation constant (pK): see (Giosa, Zadek, et al., 2024) and Appendix A.

The sO_2_‐dependent hemoglobin charge was split into a pK‐dependent (*Z*_pK_) (Gros et al., 1981; Wolf, 2022) and a chloride‐dependent (*Z*_Cl_) (Colombo et al., 1994; Wolf, 2022) component:

(3) $$Z_{\mathrm{pK}}=0.012\cdot {\mathrm{Hb}}_{g/L}\cdot \left(1-{\mathrm{sO}}_{2}\right)$$

(4) $$Z_{\mathrm{Cl}}=1.02\cdot {\mathrm{Hb}}_{\text{mMol}/L}\cdot \left(1-{\mathrm{sO}}_{2}\right)$$

With sO_2_ expressed as a fraction. A rounded saturation coefficient (*Z*_s_) was computed summing *Z*_pK_ and *Z*_Cl_, with Hb converted to g/dL, see Appendix A:

(5) $$Z_{s}=\frac{{\mathrm{Hb}}_{g/\mathrm{dL}}}{4}\cdot \left(1-{\mathrm{sO}}_{2}\right)$$

Whole‐blood buffer power (*β*) was modified from the Clinical and Laboratory Standards Institute (CLSI) (Clinical & Laboratory Standards Institute, 2009) (*β*_CLSI_ = 1.43·Hb_(g/dL)_ + 7.7) correcting the plasma buffer power of 7.7 (referred to an albumin concentration of 45 g/L) for individuals' albumin concentration (Giosa, Camporota, & Langer, 2024) and rounding coefficients (see Appendix A):

(6) $$\beta =1.5\cdot {\left(\mathrm{Hb}+\mathrm{Alb}\right)}_{g/\mathrm{dL}}$$

#### 2.2.1 SID prediction: Extended model

An expected SID_post_ was computed accounting for pH‐dependent effects only (SID_pH_), or including pK‐dependent (SID_pH‐pK_) and Cl‐dependent (SID_pH‐pK‐Cl_) saturation effects:

(7) $${\mathrm{SID}}_{\mathrm{pH}}={\mathrm{SID}}_{\mathrm{pre}}-∆Z_{\mathrm{pH}}$$

(8) $${\mathrm{SID}}_{\mathrm{pH}-\mathrm{pK}}={\mathrm{SID}}_{\mathrm{pre}}-∆Z_{\mathrm{pH}}-∆Z_{\mathrm{pK}}$$

(9) $${\mathrm{SID}}_{\mathrm{pH}-\mathrm{pK}-\mathrm{Cl}}={\mathrm{SID}}_{\mathrm{pre}}-∆Z_{\mathrm{pH}}-∆Z_{\mathrm{pK}}-∆Z_{\mathrm{Cl}}$$

#### 2.2.2 SID prediction: Simplified model

A simpler equation for the expected SID_post_ used the whole‐blood buffer power (*β*) to calculate pH‐dependent effects (Giosa, Zadek, et al., 2024):

(10) $${\mathrm{SID}}_{\beta }={\mathrm{SID}}_{\mathrm{pre}}-\beta \cdot ∆\mathrm{pH}$$

Additionally, ∆*Z*_s_ was included to account for sO_2_‐dependent effects:

(11) $${\mathrm{SID}}_{\beta -s}={\mathrm{SID}}_{\mathrm{pre}}-\beta \cdot ∆\mathrm{pH}-∆Z_{s}$$

The simplified expected post‐pre change in SID could therefore be written as follows:

(12) $$∆{\mathrm{SID}}_{\beta -s}=-\left[1.5\cdot {\left(\mathrm{Hb}+\mathrm{Alb}\right)}_{g/\mathrm{dL}}\cdot ∆\mathrm{pH}+\frac{{\mathrm{Hb}}_{g/\mathrm{dL}}}{4}\cdot ∆{\mathrm{sO}}_{2}\right]$$

### 2.3 Statistical analysis

Variables' distribution, assessed with histograms and Q‐Q plots, was non‐normal. Therefore, descriptive statistics are presented as median [IQR]. Linear mixed‐effects models (LMMs) with single subjects as random effects were used to investigate the association of ∆CO_2_ tension (∆PCO_2_), ∆sO_2_, and *β* with ∆SID. LMMs were also used to describe post‐pre changes in blood gases, with fixed effects (reported as mean and 95% CI) compared between different dilutions using interaction terms. Bland–Altman analysis was employed to describe the agreement (expressed as mean bias [limits of agreement, LoA]) between measured SID_post_ and its expected value calculated as SID_pH_, SID_pH‐pK_, SID_pH‐pK‐Cl_, SID_β_, or SID_β‐s_. Agreement was considered acceptable if the bias was within ±1 mEq/L and the maximal difference was <3 mEq/L, as previously proposed (Giosa, Zadek, et al., 2024; Story et al., 2002). The proportionality of biases and their dependence on ∆sO_2_ were assessed with LMMs. Two‐tailed *p* < 0.05 was considered statistically significant. Analyses were performed using R version 4.5.1.

## 3 RESULTS

### 3.1 Determinants of SID changes

Upon equilibration of venous blood with room‐air, the sO_2_ fraction increased by 0.46 [0.39–0.54], while PCO_2_ decreased by 29 [25–32] mmHg and pH increased by 0.25 [0.19–0.32] units. This was associated with a plasma SID decrease of 5.3 [4.2–5.6] mEq/L (all *p* < 0.01) reflecting both decreased strong cation and increased strong anion concentrations (Table 1). Notably, the plasma SID decrease was more pronounced at lower levels of dilution (Table 1).

**TABLE 1:** Changes in electrolytes upon oxygenation and decarboxylation.

Sample	No dilution		Dilution 1		Dilution 2		*p* ^a^	*p* ^b^
	Pre	Post	Pre	Post	Pre	Post		
Hb, g/dL	14.6 [13.5, 14.3]	14.6 [13.5, 15.2]	9.9 [8.8, 11.1]	9.8 [8.8, 10.9]	6.3 [5.5, 7.3]	6.3 [5.4, 7.1]	0.86	0.99
Alb, g/L	44.4 [41.6, 46.3]	44.4 [41.6, 46.3]	24.0 [21.6, 28.4]	24.0 [21.6, 28.4]	14.0 [10.2, 15.7]	14.0 [10.2, 15.7]	>0.99	>0.99
PCO_2_, mmHg	48 [46, 51]	23 [20, 25]	48 [45, 52]	21 [19, 23]	51 [48, 56]	20 [18, 22]	<0.01	0.10
pH	7.37 [7.36, 7.39]	7.55 [7.53, 7.60]	7.37 [7.35, 7.38]	7.60 [7.58, 7.62]	7.32 [7.31, 7.36]	7.66 [7.63, 7.69]	<0.01	<0.01
sO_2_, %	45 [39, 55]	94 [90, 98]	48 [42, 58]	98 [97, 98]	56 [44, 64]	98 [98, 99]	<0.01	<0.01
Na^+^, mEq/L	140 [138, 140]	137 [136, 138]	141 [140, 141]	139 [138, 140]	141 [141, 142]	140 [139, 140]	<0.01	0.09
K^+^, mEq/L	4.1 [3.9, 4.3]	4.1 [3.9, 4.2]	2.3 [2.1, 2.7]	2.3 [2.1, 2.7]	1.4 [1.0, 1.5]	1.4 [1.0, 1.5]	0.74	0.71
Ca^2+^, mMol/L	1.18 [1.17, 1.20]	1.08 [1.05, 1.10]	0.82 [0.69, 0.80]	0.73 [0.69, 0.80]	0.59 [0.48, 0.62]	0.53 [0.42, 0.55]	<0.01	0.10
Cl^−^, mEq/L	105 [104, 106]	109 [107, 109]	109 [107, 110]	111 [110, 113]	111 [110, 112]	113 [112, 114]	<0.01	0.27
Lac^−^, mEq/L	1.1 [0.9, 1.4]	1.5 [1.4, 1.8]	0.7 [0.5, 0.8]	0.9 [0.7, 1.1]	0.4 [0.3, 0.5]	0.5 [0.4, 0.6]	<0.01	<0.01
SID, mEq/L	39.4 [38.6, 41.9]	33.0 [32.4, 34.6]	34.9 [33.8, 36.7]	30.2 [29.1, 31.1]	32.3 [30.5, 33.3]	28.2 [27.6, 29.0]	<0.01	0.03

Abbreviations: Alb, albumin concentration; Ca^2+^, ionized calcium; Cl^−^, chloride; Hb, hemoglobin; K^+^, potassium; Lac^−^, lactate; Na^+^, sodium; PCO_2_, carbon dioxide tension; SID, strong ion difference; sO_2_, hemoglobin oxygen saturation fraction.

^a^ Pre versus Post sample.

^b^ Interaction (Sample*Dilution) in linear mixed‐effect models.

Changes in sO_2_ and PCO_2_, and the whole‐blood buffer power *β* were all independently associated with ∆SID (Table 2).

**TABLE 2:** Effects of oxygenation, decarboxylation and buffer power (*β*) on ∆SID.

	Slope	95% CI	*p* ^a^
∆sO_2_, fraction	−3.0	−5.4 to −0.67	**0.01**
∆PCO_2_, mmHg	0.13	0.09 to 0.16	**<0.01**
*β*, mEq/L	−0.19	−0.21 to −0.17	**<0.01**

*Note*: Values in bold indicate statistical significance at *p* < 0.05.

^a^ Relative to the slope of the linear mixed‐effects model evaluating the independent effect of each variable on ∆SID, Model = ∆SID ~ ∆sO_2_ + ∆PCO_2_ + *β* + (1|individual subject). ∆SID, post‐pre change in plasma strong ion difference; ∆sO_2_, post‐pre change in hemoglobin oxygen saturation fraction; ∆PCO_2_, post‐pre change in carbon dioxide tension; *β*, whole‐blood buffer power.

### 3.2 Prediction of SID changes

The extended model underestimated plasma SID changes when only pH effects were considered (SID_pH_), with bias worsening with ∆sO_2_. Sequential inclusion of the pK‐ and chloride‐dependent saturation effects (SID_pH‐pK_ then SID_pH‐pK‐Cl_) progressively moved the mean bias closer to *zero*, narrowed the limits of agreement, and eliminated the ∆sO_2_‐dependence (Figure 1).

**FIGURE 1:**
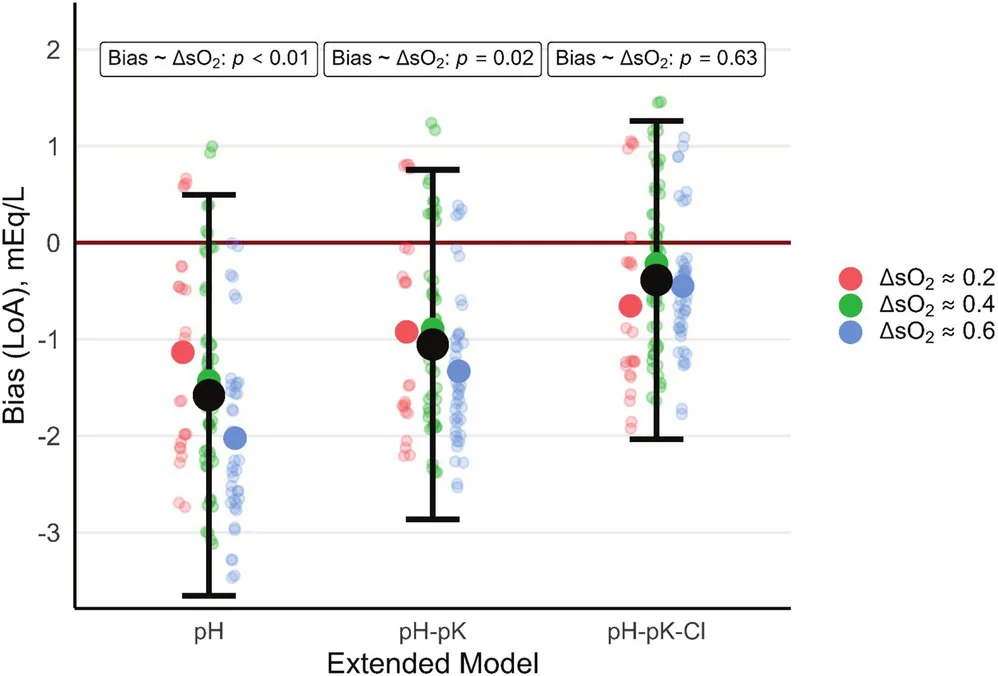
Performance of the extended model in predicting SID changes. The figure reports mean biases (black dots) and limits of agreement (LoA, black bars) of the Bland–Altman analyses investigating the ability of our extended model to predict SID_post_. The expected SID_post_ was computed accounting for pH effects only, pH effects + pK‐related saturation effects, and pH effects + pK‐related + Cl‐related saturation effects. As shown, the bias progressively approached *zero* and LoA narrowed upon addition of saturation effects: Bias(LoA) were −1.6 [−3.7 to 0.5] mEq/L, −1.1 [−2.9 to 0.8] mEq/L, and −0.4 [−2.0 to 1.3] mEq/L, respectively. The figure also reports the effect of hemoglobin saturation on the biases. As shown, unless both the pK‐ and Cl‐related effects are accounted for, the mean bias significantly worsens with increasing ∆sO_2_. Note that although the figure highlights tertiles of ∆sO_2_ (in red, green and blue colors) for graphical clarity, the effect of ∆sO_2_ on the bias specified by the *p* values was tested with linear mixed‐effects models across all measured ∆sO_2_ at a single patient level: Model = bias~∆sO_2_+ (1|individual subject).

Similarly, the simplified model narrowed the LoA upon inclusion of saturation effects, yet slightly overestimating changes in SID: mean bias went from −0.3 [−2.4 to 1.7] mEq/L for SID_β_ to 0.8 [−0.9 to 2.4] mEq/L for SID_β‐s_. Importantly, the bias became ∆sO_2_‐independent (*p* < 0.01 vs. *p* = 0.18) and no longer showed the proportionality observed with the extended model (Figure 2).

**FIGURE 2:**
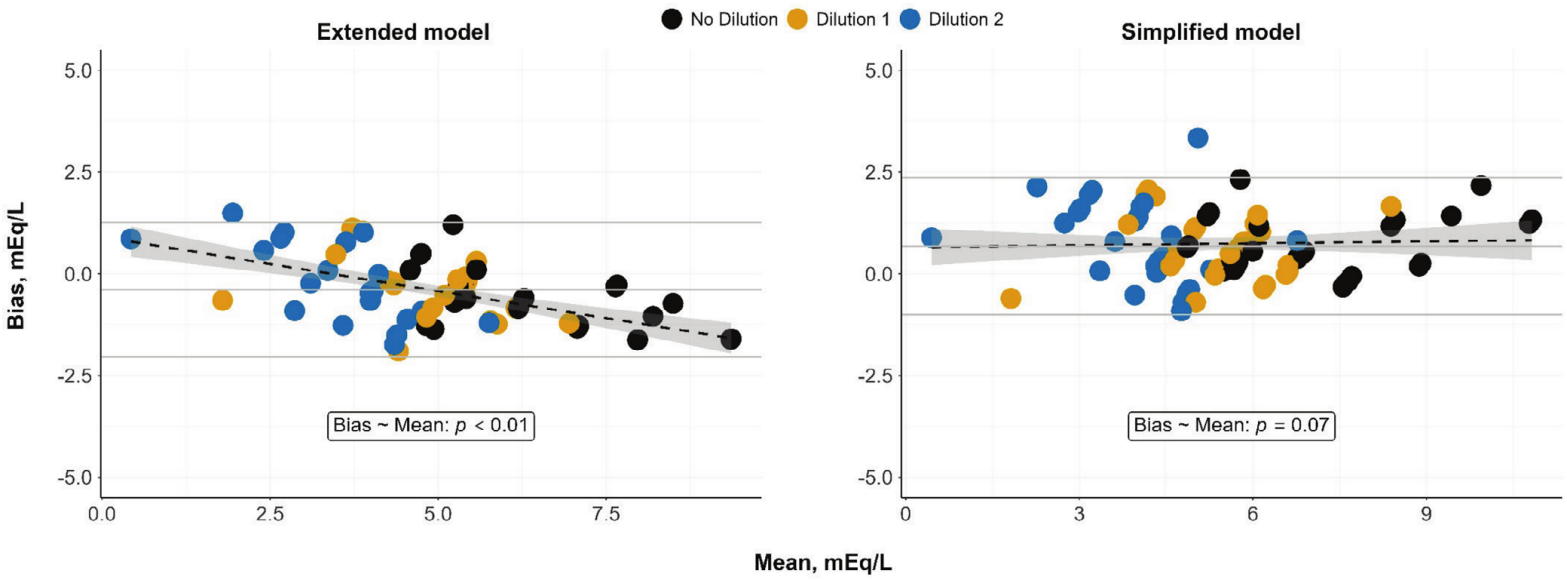
Extended versus Simplified models. The figure reports Bland–Altman analyses investigating how the extended and simplified models predict changes in SID. Mean refers to (expected + measured SID_post_)/2, bias to (expected ‐ measured SID_post_). The expected SID_post_ is SID_pH‐pK‐Cl_ for the extended model, and SID_β‐s_ for the simplified model. As shown, bias (LoA) is well within the clinically acceptable range for both the extended (−0.4 [−2.0 to 1.3] mEq/L) and simplified model (0.8 [−0.9 to 2.4] mEq/L). Notably, the simplified model overestimates ∆SID but eliminates the proportional bias that characterizes the extended model.

## 4 DISCUSSION

It has long been recognized that changes in sO_2_ are accompanied by electrolytes redistribution in humans, driven by the Haldane effect (Christiansen et al., 1914; Prange et al., 2001; Westen & Prange, 2003). However, a quantitative assessment of the resulting changes in plasma SID has been lacking. In clinical settings, significant sO_2_ variations are generally accompanied by PCO_2_ changes, as seen during the passage of blood through tissue capillaries or through the native and artificial lung (Tomarchio et al., 2024). Similarly, during primary respiratory disorders, arterial blood desaturation is often accompanied by PCO_2_ alterations, either compensatory or disease‐driven (Madotto et al., 2020). Accordingly, we investigated the combined effect of ∆sO_2_ and ∆PCO_2_ on plasma SID.

As expected, both variables were independently associated with changes in SID. This finding is consistent with recent observations by Valsecchi et al. (Valsecchi et al., 2026), who reported comparable coefficients for the effects of oxygen saturation and PCO_2_. Our study extends these observations in several important ways. First, by employing an experimental design involving sequential blood dilution, we were able to demonstrate the independent contribution of whole‐blood buffer power (*β*) to SID changes (Table 2). This was not explored by Valsecchi et al., who only investigated undiluted blood from healthy subjects with normal hemoglobin and albumin concentrations. Notably, the buffering properties of these proteins also offer a mechanistic explanation for the non‐linear relationship between ∆PCO_2_ and ∆SID reported in that study: as PCO_2_ increases, pH approaches the dissociation constant of histidine residues (pK ~ 6.75), thereby increasing buffer power and limiting further changes in pH and, consequently, in SID.

*Second*, rather than limiting our analysis to associations, we used these physiological determinants to construct predictive equations for SID changes. This was made possible by our work quantifying isolated pH effects on SID (Giosa, Zadek, et al., 2024) together with prior studies describing saturation‐dependent effects (Colombo et al., 1994; Gros et al., 1981), from which pK‐ and chloride‐dependent saturation coefficients had been previously derived (Wolf, 2022). Our results show that, during combined oxygenation and decarboxylation across a wide range of hemoglobin and albumin concentrations, ΔSID is predictable with high accuracy. Over a range of ΔSID approaching 10 mEq/L, the maximal discrepancy between predicted and measured values was approximately 2 mEq/L (Figure 2), which is within expected analytical variability for electrolyte measurements (Jones, 1993). Importantly, inclusion of saturation‐dependent effects, both pK‐ and chloride‐mediated, was necessary to maximize accuracy and eliminate systematic bias related to changes in sO_2_.

*Third*, we provide an equation that simplifies the prediction of ∆SID by using albumin and hemoglobin concentrations and a rounded saturation coefficient, possibly enabling clinical application. This simplified model, derived from the CLSI whole‐blood buffer power (*β*_CLSI_), not only maintained acceptable accuracy while avoiding complex mathematics, but also provides further insight worth discussing. Compared with the extended model, the simplified approach eliminates proportional bias but tends to slightly overestimate ΔSID, albeit within acceptable limits (Figure 2). Both phenomena are expected: on the one hand, *β*_CLSI_ uses hemoglobin and albumin as surrogates for the full red‐cell and plasma buffer powers, which also include 2,3 DPG and inorganic phosphate (Siggaard‐Andersen, 1974). Conversely, phosphate species were not included in our extended model; therefore, their contribution was likely underestimated, particularly at higher hemoglobin and albumin concentrations, explaining the progressive underestimation of ΔSID at lower levels of dilution (Figure 2); on the other hand, *β*_CLSI_ was originally derived at pH 7.4 (Siggaard‐Andersen, 1974) and is assumed to be pH‐invariant. However, in the alkalotic range of our experiment, the buffer power progressively decreases as pH moves away from the pK of buffer species (Giosa et al., 2023). This effect is captured by our extended model, but not by *β*_CLSI_, which therefore leads to a modest overestimation of ∆SID in the simplified model.

Finally, the rounded saturation coefficient deriving from the combination of pK‐ and chloride‐dependent effects (Hb_g/dL_/4·∆sO_2_) closely aligns with coefficients previously proposed by Siggaard‐Andersen (Siggaard‐Andersen, 1964) and later adopted by Zander (Lang & Zander, 2002) to correct whole‐blood base‐excess for measured sO_2_. This supports the validity of our calculations and informs the ongoing debate regarding artero‐venous differences in base excess and SID (Gattinoni & Busana, 2019; Ziegenfuß & Zander, 2019).

## 5 CLINICAL IMPLICATIONS

Our group has recently shown that incorporating pH‐dependent SID changes into bedside acid–base algorithms improves the understanding of *arterial* blood gases, particularly with regard to base‐excess interpretation (Krbec et al., 2026). However, especially in emergency medicine, initial clinical assessment is often based on *venous* blood gas analysis, where measured sO_2_ can differ substantially from arterial values. Therefore, predicting sO_2_‐dependent SID changes has the potential to allow for accurate acid–base diagnosis before arterial catheterization is established. An example is given in the Appendix (Figure A1), where a venous sample from a real clinical case is analyzed. As shown, only when both pH‐ and sO_2_‐dependent redistribution mechanisms are considered a correct interpretation of the acid–base status is possible, as confirmed by the arterial blood gas taken at a later stage. Conversely, neglecting electrolyte shifts leads to misdiagnosis, with possibly negative impact on clinical management. Notably, our simplified model allows calculations to be performed with mental arithmetic, facilitating bedside applicability.

## 6 LIMITATIONS

This study has limitations worth mentioning. *First*, some analytes were not measured, such as magnesium, which might have changed its ionized fraction therefore altering plasma SID. However, assuming changes in ionized magnesium similar to those observed in ionized calcium, our model would underestimate changes in plasma SID by less than approximately 0.2 mEq/L. Similarly, phosphate species were not included in our extended model, which may have led to a slight underestimation of SID changes. *Second*, the use of a common buffer coefficient for albumin and hemoglobin in our simplified model might introduce some inaccuracies in case of extreme imbalances in the concentrations of these two proteins. *Third*, the individual contributions of pK‐ and chloride‐dependent components of the Haldane effect were inferred from prior healthy human investigations, therefore extrapolation to disease might not systematically apply. *Finally*, while our in‐vitro model reflects in‐vivo arterialization of venous blood (Figure A1), global blood desaturation, such as in the context of respiratory failure, might lead to dilution of SID into the total extracellular space, with likely lower observed plasma changes (Krbec et al., 2026).

## 7 CONCLUSION

The contribution of hemoglobin oxygen saturation to plasma‐erythrocyte electrolyte shifts can be quantified through the Haldane effect. A simple model (Equation 12) accounting for both sO_2_‐ and pH‐dependent mechanisms enables accurate prediction of redistribution‐related plasma SID changes across a wide physiological range. More detailed models may be required under extreme acid–base conditions.

## AUTHOR CONTRIBUTIONS

**Lorenzo Giosa:** Conceptualization; data curation; formal analysis; funding acquisition; methodology; project administration; resources; software; validation; visualization. **Martin Krbec:** Conceptualization; formal analysis; funding acquisition; investigation. **Jakub Halamík:** Data curation; formal analysis; funding acquisition; investigation. **Adrian Bolnberger:** Data curation; formal analysis; funding acquisition; investigation. **Mattia Busana:** Conceptualization; formal analysis; funding acquisition; investigation. **Serena Brusatori:** Conceptualization; formal analysis; funding acquisition; investigation. **Tue Diemer:** Conceptualization; data curation; formal analysis; funding acquisition; investigation; methodology. **Søren R. Kristensen:** Conceptualization; data curation; formal analysis; funding acquisition; investigation; methodology; project administration; supervision. **Micah L. A. Heldeweg:** Conceptualization; formal analysis; funding acquisition; investigation. **Francesco Zadek:** Conceptualization; formal analysis; funding acquisition; investigation. **Stephen E. Rees:** Conceptualization; data curation; formal analysis; funding acquisition; investigation; methodology; project administration; resources; supervision. **František Duška:** Conceptualization; data curation; formal analysis; funding acquisition; investigation; project administration; supervision.

## FUNDING INFORMATION

Funded by PRIMUS/26/MED/009 programme of Charles University, and grant NW26‐01‐00360 of Czech Health Research Council (AZV).

## CONFLICT OF INTEREST STATEMENT

No conflict of interest, financial or otherwise, are declared by the authors.

## ETHICS STATEMENT

The original study was conducted in accordance with the Declaration of Helsinki, it was approved by the ethical committee of the North Jutland Region of Denmark (reference number N‐20100093), and informed written and oral consent was obtained from all subjects.

## Figures and Tables

**FIGURE A1:**
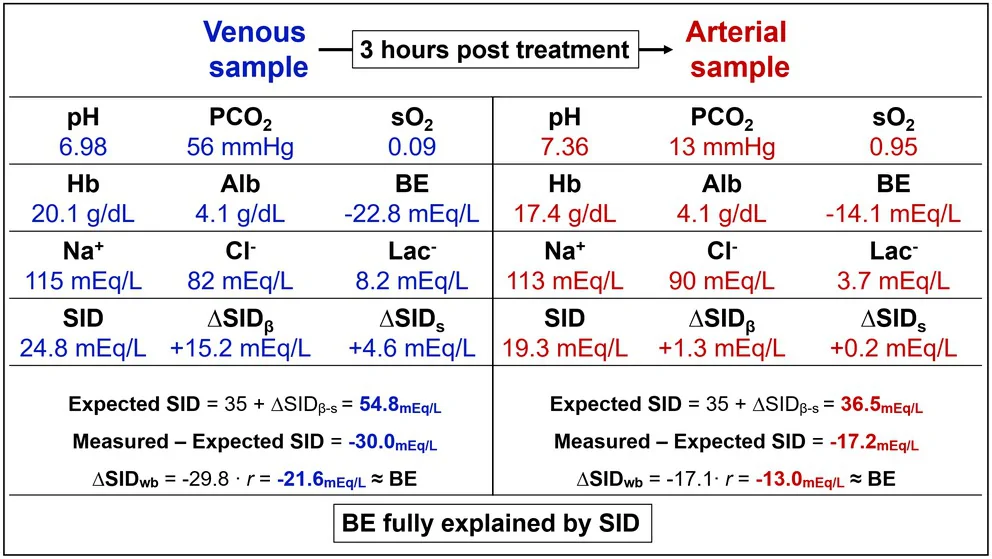
Model application to venous versus arterial blood gas interpretation.

## Data Availability

The complete dataset is available from the corresponding author upon reasonable request.

## APPENDIX A

### A.1 Albumin titrable charges

Albumin has 16 titrable histidine residues (Figge et al., 1992) with an average dissociation constant (Ka) often considered 1.77 · 10^−7^ at 37°C (Watson, 1999), according to Reeves (Reeves, 1976), which corresponds to a pKa of 6.75. The titrable charge on albumin is given by the charge on the imidazole group of histidines, which is positive, and pH‐dependent, and can be calculated as follows (Giosa, Zadek, et al., 2024; Watson, 1999):

(A1)
$$Z_{\mathrm{pH}}^{\mathrm{Alb}}=\frac{\left[H^{+}\right]\cdot \frac{\left[\mathrm{Alb}\right]}{66.5}\cdot 16}{\mathrm{Ka}+\left[H^{+}\right]}$$

where Alb is albumin in g/L, 66.5 is its molecular weight in kilodalton, and [H^+^] in mMol/L is calculated as 10^−pH^ (plasmatic pH).

### A.2 Hemoglobin titrable charges

Hemoglobin has 26 titrable residues participating in buffering: 22 titrable histidines (of note, histidines amount to a total of 38 per tetramer, but 4 for each heme are non‐titrable) and 4 alpha‐amino groups (Tanford & Nozaki, 1966). As for albumin, Reeves Ka at 37°C (1.77 · 10^−7^) (Reeves, 1976), corresponding to a pKa of 6.75, can be used as average dissociation constant for the pH‐dependent charge on these groups (Giosa, Zadek, et al., 2024), which are responsible for the positive titrable charge on hemoglobin. Accordingly:

(A2)
$$Z_{\mathrm{pH}}^{\mathrm{Hb}}=\frac{\left[H_{i}^{+}\right]\cdot \frac{\left[\mathrm{Hb}\right]}{66.8}\cdot 26}{\mathrm{Ka}+\left[H_{i}^{+}\right]}$$

where Hb is hemoglobin concentration in g/L, 66.8 is hemoglobin tetramer molecular weight in kilodalton, [H_i_^+^] in mMol/L is 10^−pHi^, where pH_i_ is intraerythrocyte pH calculated from Funder and Wieth (Funder & Wieth, 1966) as follows:

(A3)
$${\mathrm{pH}}_{i}=7.22+0.77\cdot \left(\mathrm{pH}-7.4\right)$$

When Ka, [H^+^] and [H_i_^+^] are converted to pKa, pH and pH_i_, the sum of Equations (A1 and A2) gives the total blood titrable charge *Z*_pH_ specified in Equation (2) of the main text:

(A4)
$$Z_{\mathrm{pH}}=Z_{\mathrm{pH}}^{\mathrm{Alb}}+Z_{\mathrm{pH}}^{\mathrm{Hb}}=\mathrm{Alb}\cdot \frac{16\cdot 10^{-\mathrm{pH}}}{10^{-6.75}+10^{-\mathrm{pH}}}+\mathrm{Hb}\cdot \frac{26\cdot 10^{-\mathrm{pHi}}}{10^{-6.75}+10^{-\mathrm{pHi}}}$$

where Alb and Hb are in mMol/L.

### A.3 Rounded saturation coefficient

The rounded sO_2_‐dependent hemoglobin charge (*Z*_s_) was computed summing *Z*_pK_ and *Z*_Cl_ of Equations (3) and (4) in the main text, after converting Hb to g/dL using a tetramer molecular weight of 66.8 kilodalton:

(A5)
$$Z_{s}=\left(0.012\cdot 10\cdot {\mathrm{Hb}}_{g/\mathrm{dL}}+1.02\cdot \frac{10\cdot {\mathrm{Hb}}_{g/\mathrm{dL}}}{66.8}\right)\cdot \left(1-{\mathrm{sO}}_{2}\right)$$

This equates to:

(A6)
$$Z_{s}=\left(0.27\cdot {\mathrm{Hb}}_{g/\mathrm{dL}}\right)\cdot \left(1-{\mathrm{sO}}_{2}\right)$$

Which can be approximated to:

(A7)
$$Z_{s}=\frac{{\mathrm{Hb}}_{g/\mathrm{dL}}}{4}\cdot \left(1-{\mathrm{sO}}_{2}\right)$$

### A.4 Modified whole‐blood buffer value (*β*)

The whole‐blood buffer power (*β*) is commonly estimated according to the Clinical and Laboratory Standards Institute (CLSI) (Clinical & Laboratory Standards Institute, 2009):

(A8)
$$\beta_{\text{CLSI}}=1.43\cdot {\mathrm{Hb}}_{g/\mathrm{dL}}+7.7$$

In this formulation, the constant plasma buffer contribution (7.7) is referred to a standard albumin concentration of 4.5 g/dL. To account for inter‐individual variability in plasma protein buffering, the plasma buffer term was corrected for the measured albumin concentration, leading to:

(A9)
$$\beta =1.43\cdot {\mathrm{Hb}}_{g/\mathrm{dL}}+1.71\cdot {\mathrm{Alb}}_{g/\mathrm{dL}}$$

This equation can be re‐written, rounding both coefficients to 1.5 and adding a residual term:

(A10)
$$\beta =1.5\cdot {\mathrm{Hb}}_{g/\mathrm{dL}}+1.5\cdot {\mathrm{Alb}}_{g/\mathrm{dL}}-0.07\cdot {\mathrm{Hb}}_{g/\mathrm{dL}}+0.21\cdot {\mathrm{Alb}}_{g/\mathrm{dL}}$$

By applying the simple and physiologically sound assumption that Hb_g/dL_ ≈3·Alb_g/dL_ to the residual term, the latter sums up to *zero*; therefore:

(A11)
$$\beta =1.5\cdot {\left(\mathrm{Hb}+\mathrm{Alb}\right)}_{g/\mathrm{dL}}$$

This case was presented at the 2025 SMART meeting in Milan and refers to a patient admitted to the emergency department with confusion and a 10‐day history of profound diarrhea and poor fluid intake. Initial assessment was based on the peripheral venous sample reported on the left side of the figure. As shown, the fraction of saturated hemoglobin (sO_2_) is low and the partial pressure of carbon dioxide (PCO_2_) high, as typically observed in peripheral samples. Moreover, a severe metabolic acidosis is suggested by the markedly negative base excess (BE calculated according to Zander (Lang & Zander, 2002) using our buffer power *β* = 1.5·(Hb + Alb)_(g/dL)_ for consistency). An abbreviated apparent strong ion difference (SID) was computed from available electrolytes as sodium (Na^+^)—chloride (Cl^+^)—lactate (Lac^−^), resulting in 24.8 mEq/L, that is, ~ 10 mEq/L lower than the value of 35 mEq/L considered as reference in clinical practice (Krbec et al., 2026). This would suggest that most of the BE is not explained by SID alterations, leading to the diagnosis of unmeasured ion acidosis (Giosa, Zadek, et al., 2024). However, including electrolytes redistribution in the framework challenges such conclusion. According to our simplified model, the SID in this venous sample should be increased by 15.2 mEq/L due to pH‐induced electrolytes shifts (∆SID_β_ = 1.5·(Hb + Alb)_(g/dL)_ (7.4—pH)), plus 4.6 mEq/L due to saturation‐induced redistribution (∆SID_s_ = Hb_g/dL_/4·(1—sO_2_)). Therefore, the “normal,” expected SID should be 54.8 mEq/L rather than 35 mEq/L. Consequently, the discrepancy between measured (24.8 mEq/L) and expected (54.8 mEq/L) values is −30.0 mEq/L. Converting this plasma SID deficit into its whole‐blood equivalent (SID_wb_) via *r* = 1–0.014·Hb_g/dL_ (Giosa, Zadek, et al., 2024; Lang & Zander, 2002) leads to a whole‐blood strong ion deficit of −21.6 mEq/L, explaining the totality of BE. This patient was therefore likely suffering from a low apparent SID acidosis due to loss of water and sodium in the stools with hyperlactatemia. This was confirmed by the arterial sample (right side of the figure) obtained around 3 h post treatment initiation. As shown, metabolic acidosis was still present and fully explained by low SID, yet with reduced severity (less negative BE) as a consequence of rehydration. Note that albumin concentration was only available on admission and considered constant, although it probably decreased upon rehydration, together with Hb. However, the effect of its changes on BE was likely minor. Indeed, assuming a dilution similar to Hb in the arterial sample, albumin concentration would drop from 4.1 to 3.5 g/dL, equivalent to only ~1.8 mEq/L increase in BE (although greater changes cannot be excluded). Finally, our calculation of apparent SID does not include potassium and calcium, which were not available from this patient's records. Although our abbreviated calculation of apparent SID has been shown to provide good results in clinical practice (Krbec et al., 2026), unrecognized imbalances in potassium and calcium might mask the possible presence of unmeasured ions such as ketones in this starved patient.
